# Comparison of Oxidant/Antioxidant, Detoxification Systems in Various Tissue Homogenates and Mitochondria of Rats with Diabetes Induced by Streptozocin

**DOI:** 10.1155/2012/386831

**Published:** 2012-03-28

**Authors:** Veysel Kenan Çelık, Zeynep Deniz Şahın, İsmail Sari, Sevtap Bakir

**Affiliations:** ^1^Departments of Biochemistry, Cumhuriyet University School of Medicine, 58140 Sivas, Turkey; ^2^Department of Histology and Embryology, Cumhuriyet University School of Medicine, 58140 Sivas, Turkey

## Abstract

*Objective*. Oxidative stress is considered to be the main factor in the development of diabetic complications and tissue injury. our objective was to investigate and compare the oxidant/antioxidant conditions and detoxification mechanisms of the liver, lung, kidney, cardiac tissues, and mitochondria of rats with diabetes induced by streptozocin (STZ). *Methods*. Rats with diabetes induced by streptozocin were anesthetized by administering 90 mg/kg ketamine hydrochloride and 3 mg/kg xylazine hydrochloride. Thoracic cavities were incised open; liver, lung, kidney, and cardiac tissues were removed and stored at −70°C. All samples were homogenized and mitochondrial fractions were separated. Total Antioxidant Status (TAS), Total Oxidant Status (TOS), Oxidative Stress Index (OSI), Paraoxonase (PON), Arylesterase, Catalase (Cat), Malondialdehyde (MDA), and Glutathion-S-transferase were measured in each fraction. *Results*. MDA and TOS levels were significantly increased in liver tissues, and T OS and OSI were increased in the mitochondrial fractions of diabetic rats. These increases were not statistically significant compared to the control group. No significant differences were determined in the antioxidant and GST activities. *Conclusion*. According to our results, oxidative stress has not developed in rats with diabetes induced by streptozocin. The detoxification system was induced; however, this induction did not differ significantly from the controls.

## 1. Introduction

Diabetes is a rapidly spreading disease due to current adverse living conditions and unbalanced nutrition habits. The incidence and prevalence of type II diabetes are increasing particularly in industrializing and developing nations, and this increase is estimated to exceed 200 million as of the end of 2011 [1]. Chronic complications of diabetes affect several organ and systems creating the primary causes of morbidity and mortality. Overall, chronic complications are grouped into two categories as vascular and nonvascular complications [2–4].

Diabetes is a metabolic disease; however, it has long been known that its complications are associated with oxidative stress [5]. Chronic hyperglycemia leads to increased oxidative stress particularly in tissues where complications of diabetes develop [6]. The mechanisms that increase oxidative stress in diabetes include nonenzymatic glycation, autooxidative glycation, metabolic stress secondary to the changes in energy metabolism, activity of the sorbitol pathway, levels of inflammatory mediators, and tissue injury resulting from the changes in antioxidant defense system [7–13].

In this study, our objective was to investigate and compare the antioxidant-oxidant systems (TAS,TOS, OSI, PON, Arylesterase, Catalase, and MDA) and levels of GST enzyme that plays an important role in detoxification mechanisms in heart, liver, lung, and kidney tissues and mitochondrial fractions in rats with hyperglycemia (not chronic) developed with STZ, a diabetogenic chemical agent.

## 2. Material and Methods

### 2.1. Development of Experimental Diabetes

Twelve female and male *Wistar albino* rats of 250–300 grams in weight, reproduced in the Experimental Animals Laboratory of Cumhuriyet University, Medical Faculty were used in this study. Rats were separated into two groups as control and study groups and were provided with free access to food and water. An approval document dated November 1st; 2007 and numbered B.30.2.CUM.0.01.00.00-50/214 was obtained from the Cumhuriyet University, Experimental Animals Ethics Committee.

Blood glucose levels of rats were measured following and overnight fasting (Lever Check TD-4222). Rats with blood glucose levels 80–110 mg/dL were considered normal. A single dose of intraperitoneal 60 mg/kg streptozocin (STZ; Sigma Chemical Co., St. Louis Missouri, USA) resolved in 0.1 M citrate buffer of 4.5 pH was used to develop diabetes in the experimental group of animals without sex distinction. Blood glucose levels were measured in the intravenous blood samples obtained from the tails of rats at 48 hours of streptozocin injection. Rats with blood glucose levels of 250 mg/dL were considered diabetic.

### 2.2. Surgical Procedure

All rats were anesthetized at week two with an intramuscular injection of 90 mg/kg ketamine hydrochloride, 3 mg/kg of xylazine hydrochloride into left fore leg muscle. Thoracic cavities were incised open; liver, lung, kidney, and heart tissues were removed and stored at −70°C.

### 2.3. Mitochondria Extract

All defrosted tissues were homogenized in ice shower containing 4 mL of 0.2 M phosphate buffer at pH 7.4. Homogenates were centrifuged at 3000 g for 15 minutes at 4°C to remove tissue remnants. Activities were determined in the supernatant.

Mitochondria were extracted according to the method defined by Max et al. [14] and modified by Mousa [15]. Samples of 1 gram was obtained from each tissue and homogenized in 5 mL of 0.25 M sucrose solution. Homogenates were centrifuged at 800 g for 15 minutes at 4°C, supernatant was later recentrifuged at 20 000 g for 10 minutes. Following the removal of the supernatant, the mildly swollen layer on top of the mitochondrial pellet was removed with the gentle addition of sucrose solution. Pellet was later prepared for analyses by suspension with 1.2 mL of 0.02 M phosphate buffer at pH 7.4.

### 2.4. Activity Analyses

MDA were measured spectrophotometrically according to the thiobarbituric acid (TBA) method [16]. TAS, TOS, PON, and arylesterase activities were measured using the commercial kit (Rel Assay Diagnostic) Syncron LX autoanalyzer. OSI was calculated according to the following formula: OSI: TOS/TASX100.

Catalase activity was determined by measuring the amount of H_2_O_2_ decreased at 240 nm wavelength according to the method defined by Beers and Sizer [17]. GST activities were calculated spectrophotometrically at 340 nm according to the method defined by Habig et al. [18].

### 2.5. Statistical Analysis

Statistical analysis was performed using the Kruskal Wallis analysis (SPSS, 14.0). Paired comparisons were performed with Mann-Whitney *U* test, when a *P* value of ≤0.05 was obtained. A *P* value of ≤0.05 was considered significant.

## 3. Results

In the comparison of parameters measured in control group by tissue types as shown in Table 1 (lung, liver, kidney, and heart), no significant differences were determined in terms of MDA and PON. Paired comparisons with Mann-Whitney *U* test demonstrated that level of MDA was significantly higher in heart tissue compared to the lung and kidney tissues (*P* < 0.05), and PON activity was significantly higher in the kidney tissue compared to the liver and lung tissues (*P* < 0.05). Kruskal Wallis analysis was used to determine significant differences between the groups, and no significant differences were determined in the paired comparison of other tissue types (*P* > 0.05).

No significant differences were determined in terms of MDA and TOS between the groups in the evaluation of parameters measured in diabetic group by tissue types. Paired comparisons with Mann-Whitney *U* test demonstrated that MDA level of liver tissue was significantly higher compared to that of the heart tissue (*P* < 0.05). TOS level of liver tissue was significantly higher compared to that of the heart and lung tissues (*P* < 0.05). Renal TOS level was also significantly higher compared to the lung and heart (*P* < 0.05). Kruskal Wallis analysis was used to determine significant differences between the groups, and no significant differences were determined in the paired comparison of other tissue types (*P* > 0.05) (Table 1).

A significant difference was determined in terms of TOS, OSI (%), GST, and Catalase between the groups in the evaluation of parameters measured in mitochondrial tissue fractions by tissue types in the diabetic group, as shown in Table 2. In the paired comparison of these groups with the Mann-Whitney *U* test, TOS and OSI levels were significantly higher in the mitochondrial fraction of liver tissue compared to the heart tissue and kidney tissue, respectively (*P* < 0.05). Catalase-specific activity in the mitochondrial fraction of liver tissue was significantly higher compared to that of the mitochondrial fraction of lung tissue. GST-specific-activity was appointed to be significantly higher in the mitochondrial fraction liver tissue compared to mitochondrial fractions of all tissue types. Kruskal Wallis analysis was used to determine significant differences between the groups, and no significant differences were determined in the paired comparison of other tissue types (*P* > 0.05).

Significant difference was determined in terms of MDA, OSI (%) and Catalase between the groups in the evaluation of parameters measured in mitochondrial tissue fractions of control group by different tissue types (Table 2). Paired comparisons with Mann-Whitney *U* test in these groups demonstrated that OSI (%) level was significantly higher in mitochondrial fraction of liver tissue compared to kidney and heart tissues (*P* < 0.05). Catalase-specific activity in mitochondrial fraction of kidney tissue was significantly higher compared to that of lung tissue. MDA level was determined to be significantly higher in the mitochondrial fraction of liver tissue compared to mitochondrial fractions of all other tissue types. Additionally, MDA level of mitochondrial fraction of lung tissue was also significantly higher compared to the MDA levels in the mitochondrial fraction of kidney and heart tissues (*P* < 0.05). Kruskal Wallis analysis was used to determine significant differences between the groups, and no significant differences were determined in the paired comparison of other tissue types (*P* > 0.05).

No significant differences were determined between the diabetic and control groups in the statistical analysis of parameters measured in all tissue types (tissue-mitochondrial) (*P* > 0.05).

## 4. Discussion

Male and female rats were equally distributed to each group to prevent the influence of confounding factors including sex. The most significant increase in the level of MDA among rats with chemically induced diabetes was observed in the liver when the results were evaluated between different tissues and within the same group (Table 1). Studies on rats with STZ-induced diabetes have demonstrated that MDA levels were significantly increased in the liver [19] and kidney mitochondria [19, 20]. According to our results this increase was limited to the liver; however it was not statistically significant. The increase in TOS in both liver and mitochondrial fractions in the diabetic group led to an increase also in the OSI. Although MDA, TOS, and OSI were increased, these were not statistically significant increases compared to the control group (*P* > 0.05). No significant differences were noted between the two groups, tissues and mitochondrial fractions when levels of TAS were compared.

A significant increase only in the catalase activity of liver mitochondria of the diabetic group was noted in the comparison of the antioxidative enzymes PON, arylesterase, and catalase (*P* = 0.03). The lack of difference in OSI levels in between the groups suggested us the contribution of the increased catalase activity. A study on acute diabetic rats has demonstrated that catalase activity is not altered [21]; however another time-dependent study has demonstrated an increase in the first week followed by a decrease compared to the controls [22]. In the long term, this will affect the OSI and trigger oxidative injury. Planning of a time-dependent study is important to demonstrate the alterations in OSI.

On the other hand, in terms of the data of GST, liver mitochondrial fraction was increased in the diabetic group, although not statistically significantly compared to the controls. This increase in GST is in response to STZ and causes a reflex in the detoxification system. It is known that medications and endogenous-exogenous chemical substances are first metabolized by the liver microsomal oxidase systems leading to the formation of several toxic intermediate products. This is the phase 1 reaction needed for the metabolism of xenobiotics. These toxic substances are conjugated and transformed in to polar compounds that dissolve in water in order to excrete in urine in phase 2 reactions. GST is one of the most important phase 2 systems [23]. The increase in GST activity will both prevent the cells from the toxic effects of harmful compounds and prevent the development of oxidative injury. The lack of difference in terms of OSI in between the groups possibly resulted from the increased GST activity. Although we did not find any other study investigating the relationship between GST and diabetes, there is one study on the effect of STC on GST. This study has suggested that GST activity was decreased [24]. The lack of more studies on this topic is a limitation in terms of the comparison of our results. Further studies on this topic will be very important to remove the uncertainties, since the reported result contradicts with ours.

Consequently, although partial differences were observed between the tissues within the same group, no statistically significant difference was determined between the tissues and mitochondrial fractions. However, oxidative stress might develop in the long term in a chronic picture of diabetes and lead to tissue injury in addition to reduction in catalase and GST activity.

## 5. Conclusion

Consequently, although partial differences have been noted in both oxidant/antioxidant systems and detoxification enzyme (i.e., GST) activity in tissue and mitochondria of rats with experimental diabetes, these differences are not statistically significant compared to the control group. According to our results, oxidative stress has not developed in acute diabetes induced by a chemical agent, suggesting that oxidative stress might develop in long term and lead to tissue injury in a chronic picture of diabetes.

## Figures and Tables

**Table 1:** Evaluation of parameters measured in both group by tissue types.

Measurement	Lung X ± S	Liver X ± S	Kidney X ± S	Heart X ± S
MDA (nmol/mL)				
Control	3,81 **± **1,1**^§^**	3,22 **± **0,7	5,22 **± **0,8**^*ξ*^**	1,29 **± **0,4**^*ξ*^**
Diabetic	3,22 ± 3,1	9,01 ± 7,8*	3,30 ± 1,5	0,50 ± 0,4*

TAS (mmol trolox Equiv/L)				
Control	0,423 **± **0,54	0,406 **± **0,21	0,376 **± **0,10	0,143 **± **0,03
Diabetic	0,19 ± 0,06	0,53 ± 0,28	0,45 ± 0,28	0,18 ± 0,04

TOS (*μ*mol H_2_O_2_ Equiv/L)				
Control	7,75 **± **10,4	4,46 **± **1,2	6,40 **± **2,4	2,53 **± **0,9
Diabetic	2,22 ± 0,03^#^	6,29 ± 1,05^#^	4,59 ± 0,78**^#^**	1,49 ± 0,6^**#**^

OSİ (%)				
Control	16,83 **± **2,35	13,12 **± **6,70	16,97 **± **4,12	17,35 **± **3,42
Diabetic	12,05 ± 3,4	15,82 ± 11,4	12,49 ± 6,99	8,07 ± 1,44

PON (U/L)				
Control	4,33 **± **3,2*	4,33 **± **1,5*	9,33 **± **0,5*	7,66 **± **1,5
Diabetic	6,00 ± 3,0	6,00 ± 4,3	8,00 ± 1,0	7,33 ± 1,5

Arylesterase (U/L)				
Control	4365,0 **± **410,7	4347,6 **± **738,5	4093,3 **± **359,1	4068,3 **± **380,6
Diabetic	4785,0 ± 111,8	4088,3 ± 541,0	4350,0 ± 172,4	4249,0 ± 483,0

GST (U/mgPrt)				
Control	0,0032 **± **0,002	0,0547 **± **0,040	0,0073 **± **0,004	0,0062 **± **0,003
Diabetic	0,0011 ± 0,001	0,0238 ± 0,020	0,0020 ± 0,003	0,0173 ± 0,029

Catalase (U/mgPrt)				
Control	0,545 **± **0,07	0,1176 **± **0,04	0,0371 **± **0,02	0,0188 **± **0,01
Diabetic	0,012 ± 0,003	0,0665 ± 0,029	0,0381 ± 0,036	0,0488 ± 0,04

*P = 0.05, ^§^ *P* = 0,03, ^#^ *P* = 0.01.

**Table 2:** Evaluation of parameters measured in the mitochondrial fraction of both group by tissue types.

Measurement	Lung X ± S	Liver X ± S	Kidney X ± S	Heart X ± S
MDA (nmol/L)				
Control	4,23 ± 0,59^#^	6,04 ± 0,12^#^	2,54 ± 0,67^#^	1,21 ± 0,29^#^
Diabetic	2,90 ± 2,00	6,21 ± 4,63	4,43 ± 3,81	1,61 ± 0,99

TAS (mmol trolox Equiv/L)				
Control	0,79 ± 0,86	0,69 ± 0,55	1,46 ± 0,32	0,83 ± 0,59
Diabetic	0,51 ± 0,10	2,11 ± 1,65	1,28 ± 0,20	0,42 ± 0,15

TOS (*μ*mol H_2_O_2_ Equiv/L)				
Control	8,83 ± 11,79	7,82 ± 9,31	2,27 ± 0,79	1,57 ± 1,28
Diabetic	1,87 ± 0,35	10,00 ± 7,09**^∆^**	2,19 ± 0,76	0,85 ± 0,33

OSİ (%)				
Control	8,66 ± 5,03	16,10 ± 21,67^§^	1,52 ± 0,28**^§^**	1,82 ± 0,32^§^
Diabetic	3,84 ± 1,44	5,59 ± 2,15*	1,68 ± 0,22*	2,20 ± 1,0

PON (U/L)				
Control	6,00 ± 2,00	11,66 ± 12,50	7,33 ± 2,08	8,00 ± 1,00
Diabetic	7,33 ± 0,57	10,66 ± 5,85	7,33 ± 1,52	9,66 ± 2,88

Arylesterase (U/L)				
Control	5006,6 ± 742,0	4330,6 ± 951,4	4636,3 ± 210,7	3996,3 ± 183,9
Diabetic	3583,6 ± 1487,6	4460,6 ± 449,5	4348,0 ± 165,3	3996,6 ± 136,0

GST (U/mgPrt)				
Control	0,0027 ± 0,001	0,0307 ± 0,016	0,0063 ± 0,004	0,0077 ± 0,005
Diabetic	0,0023 ± 0,0007**^∆^**	0,0272 ± 0,003**^∆^**	0,0038 ± 0,001**^∆^**	0,0087 ± 0,004**^∆^**

Catalase (U/mgPrt)				
Control	0,0126 ± 0,004**^*ξ*^**	0,0655 ± 0,032	0,1005 ± 0,047**^*ξ*^**	0,0254 ± 0,02
Diabetic	0,0086 ± 0,003**^§^**	0,0575 ± 0,024**^§^**	0,0429 ± 0,0094	0,0266 ± 0,009

^#^ *P* = 0.01, **^∆^** *P* = 0.02, **^§^** *P* = 0.03, **^*ξ*^** *P* = 0.04, *P = 0.05.
